# The Double Burden of Food Insecurity and Poor Oral Health Among Pregnant Women in eThekwini, KwaZulu‐Natal

**DOI:** 10.1155/ijod/9192355

**Published:** 2026-09-08

**Authors:** Sinegugu Shongwe, David Seidenfeld, Themba Ginindza

**Affiliations:** ^1^ Discipline of Public Health, School of Medicine, College of Health Sciences, University of KwaZulu-Natal, Durban, KwaZulu-Natal, South Africa, ukzn.ac.za; ^2^ American Institutes for Research, Washington DC, USA; ^3^ Cancer and Infectious Diseases Epidemiology Research Unit (CIDERU), Discipline of Public Health, School of Medicine, University of KwaZulu-Natal, Durban, KwaZulu-Natal, South Africa, ukzn.ac.za

**Keywords:** antenatal care, food insecurity, oral health, pregnancy, South Africa

## Abstract

**Objectives:**

To estimate the prevalence of household food insecurity and of poor oral health, and to identify factors associated with each outcome among pregnant women attending public‐sector antenatal care (ANC) at two community health centres (CHCs) in eThekwini, KwaZulu‐Natal (KZN), South Africa.

**Methods:**

An analytic cross‐sectional study was conducted among 420 pregnant women attending ANC at Cato Manor and KwaMashu CHC. Data were collected from February to March 2026, using interviewer‐administered structured questionnaires. Food insecurity was measured using the eight‐item Food Insecurity Experience Scale (FIES), while oral health was assessed using adapted WHO Oral Health Survey items. Logistic regression was used for statistical analysis.

**Results:**

Reported food insecurity prevalence was 44.4% (95% CI: 39.7–49.2), with 20.0% (95% CI: 16.5–24.2) reporting moderate or severe food insecurity. Poor oral health affected 12.6% (95% CI: 9.8–16.2) and 57.0% (95% CI: 52.2–61.7) reported at least one pregnancy‐onset symptom. In bivariate analysis, moderate or severe food insecurity was more common among younger women (*p* = 0.005), those in the lowest income bracket (*p* = 0.010), those who brushed once daily (*p* = 0.025), and those who never visited a dentist (*p* = 0.029). After multivariable adjustment, moderate or severe food insecurity remained independently associated with poor oral health (aOR 5.11; 95% CI: 2.72–9.58; *p*  < 0.001).

**Conclusions:**

Household food insecurity was significantly and independently associated with poor oral health among pregnant women attending ANC. As a cross‐sectional study, this study demonstrates association rather than causation and supports the need for longitudinal or prospective research to establish causal pathways. They also support integrating food insecurity screening into ANC and inform maternal health, nutrition, and oral health policies to strengthen comprehensive maternal healthcare services in South Africa.

## 1. Introduction

Maternal oral health is often overlooked in antenatal care (ANC) despite increasing evidence that pregnancy significantly affects oral physiology and that inadequate maternal dental health correlates with negative pregnancy outcomes, such as preterm birth and low birthweight [1]. Hormonal fluctuations during pregnancy, especially heightened levels of oestrogen and progesterone, augment gingival vascular permeability and the inflammatory reaction to dental plaque, resulting in gestational gingivitis affecting an estimated 60%–75% of pregnant women globally [2, 3]. Xerostomia (dry mouth) induced by pregnancy, changes in salivary content, and dietary cravings exacerbate the risk of oral disease [4]. Periodontal disorders currently impact around 796 million individuals worldwide, ranking as the sixth most common non‐communicable oral disease [5]. Pregnancy may increase vulnerability to food insecurity as rising nutritional requirements coincide with household resource pressures, particularly in socioeconomically disadvantaged settings [6]. A previous study conducted at antenatal clinics in KwaZulu‐Natal (KZN), investigating the oral health status and treatment needs of pregnant women, reported that pregnancy epulis was diagnosed in 38 (8.5%), oral lesions in 65 (14.7%), and tooth mobility in 26 (5.9%) mothers but did not examine food insecurity as an exposure [7]. The 2024 WHO Bangkok Declaration clearly designates oral diseases as a global public health priority that requires integration with broader non‐communicable disease and universal health coverage initiatives [8]. Despite this recognition, maternal oral health is inadequately incorporated into standard prenatal care in the majority of low‐ and middle‐income contexts, including South Africa.

The Food and Agriculture Organisation (FAO) defines household food insecurity as the lack of regular access to sufficient, safe, and nutritious food for normal growth, development, and an active and healthy life. It is among the most pressing global social determinants of health and may shape oral health risk through several plausible pathways [9, 10]. The 2025 State of Food Security and Nutrition in the World report estimated that 2.3 billion people, ~28% of the global population, encountered moderate or severe food insecurity, with women disproportionately impacted across all regions [11]. The burden is significantly disproportionate among regions. In Sub‐Saharan Africa, the prevalence of moderate or severe food insecurity reached 58.9% in 2024, exceeding the global average by more than twofold, and hunger impacted over one in five Africans, with an estimated 23% of pregnant women in Africa being malnourished [12]. The 2024 General Household Survey in South Africa revealed that almost 22% of families experienced inadequate or severely inadequate access to food, affecting nearly 14 million individuals who endure daily hunger [13]. KZN is among the provinces most affected by food insecurity in SA [14]. Previous reports have shown higher levels of food insecurity among female‐headed households, which constitute ~46.8% of households in the province, placing many women and children at increased risk of inadequate access to food [14].

Food insecurity may influence oral health through several plausible pathways: reduced dietary diversity and inadequate intake of micronutrients required for periodontal tissue integrity [15, 16]; greater reliance on inexpensive, energy‐dense, sugar‐rich foods; delayed or forgone dental care due to competing financial demands; and heightened psychosocial stress that can affect oral health behaviours [17, 18]. These pathways are especially salient in pregnancy when nutritional requirements rise.

**Figure 1:**
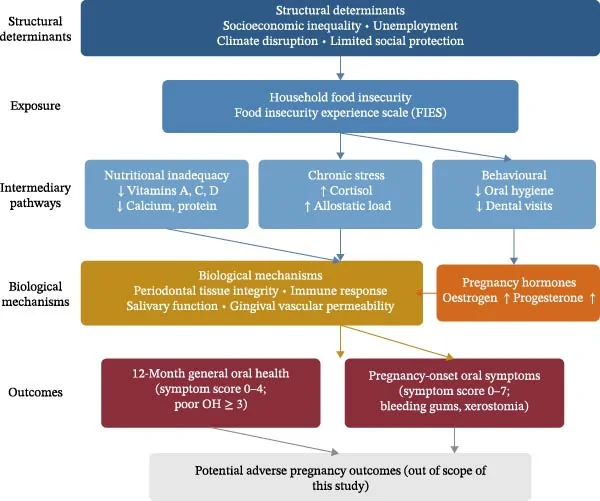
Conceptual framework linking food insecurity to oral health during pregnancy. The framework synthesises the WHO Social Determinants of Health framework (Solar & Irwin [23]), Petersen’s common risk factor approach (2003), life course epidemiology (Kuh & Ben‐Shlomo [26]), and Seligman & Schillinger’s hunger‐disparity model (2010).

International studies have demonstrated significant correlations between food insecurity and oral health in non‐pregnant adults [16, 19]. During pregnancy, the co‐occurrence of food insecurity and poor oral health may contribute to adverse maternal and pregnancy outcomes [20]. Despite this international evidence in non‐pregnant adults, evidence on the relationship between food insecurity and oral health among pregnant women in South Africa and the broader sub‐Saharan African region remains scarce; this study addresses that gap. Addressing this gap is important for informing integrated ANC approaches aligned with the WHO Global Oral Health Action Plan (2023–2030) [21] and the Sustainable Development Goals (SDG), particularly SDG 2 (Zero Hunger) and SDG 3 (Good Health and Well‐being) [22]. Guided by an integrated conceptual framework synthesising the WHO Social Determinants of Health framework [23], Petersen’s common risk factor approach for oral health [24, 25], life course epidemiology [26], and Seligman and Schillinger’s hunger‐disparity model [18] (Figure 1). Within this framework, the WHO Social Determinants of Health model situates food insecurity within upstream structural drivers; Petersen’s common risk factor approach links food insecurity and oral disease through shared behavioural and dietary risks; life‐course epidemiology frames pregnancy as a sensitive period during which exposures may have amplified effects; and Seligman and Schillinger’s hunger‐disparity model specifies how food insecurity translates into physiological and behavioural risk. This study estimated the prevalence of household food insecurity and of poor oral health and identified factors associated with each outcome among pregnant women attending ANC at two public healthcare facilities in KZN, South Africa.

Outcomes are partitioned into a 12‐month general oral health score and pregnancy‐period changes. Of the three intermediary pathways depicted, none was measured directly in this study: nutritional inadequacy and chronic stress (cortisol and allostatic load), together with the downstream biological mechanisms, are theorised based on the cited frameworks and were not assessed, whereas behavioural pathways were partially captured through self‐reported tooth‐brushing frequency, dental visit history, and dietary‐frequency items. Distal adverse pregnancy outcomes are recognised but were neither collected nor analysed and are shown only to situate the study within the wider maternal health literature; they lie outside the scope of this study.

## 2. Methods

### 2.1. Study Area

The study was conducted in Durban, the third‐most populous city in South Africa and the largest in the province of KZN. Durban forms part of the eThekwini Metropolitan Municipality, which covers ~2556 km [2] and had an estimated population of 4.2 million in 2022 [27]. The municipality is characterised by substantial socioeconomic heterogeneity, ranging from affluent urban suburbs to densely populated townships and informal settlements. KZN province carries a high burden of poverty, unemployment, and food insecurity, with national surveys consistently identifying it as among the provinces most affected by inadequate household food access [28].

### 2.2. Study Setting

Data were collected at two community health centres (CHCs) in the eThekwini District: Cato Manor and KwaMashu CHC. Cato Manor CHC is located in Umkhumbane (Mayville), an urban community of ~93,000 residents characterised by high unemployment and low socioeconomic conditions, and provides primary health care services, including maternal and ANC, to a catchment population of ~70,000 people [29, 30]. KwaMashu CHC is situated in KwaMashu township, the second‐largest township in KZN, and serves a large peri‐urban population of ~175,000 residents with socioeconomic challenges typical of township settings [31]. Both CHCs were purposively selected because they serve populations with substantial social and health needs, making them appropriate settings for investigating food insecurity and maternal oral health among pregnant women.

### 2.3. Study Design and Population

A facility‐based, cross‐sectional analytic study was conducted at the two CHCs described above between February and March 2026. The study formed part of a broader sequential explanatory mixed‐methods study investigating food insecurity and maternal oral health among pregnant women attending ANC services. The quantitative cross‐sectional component was undertaken to determine the prevalence and associated factors of food insecurity and poor oral health during pregnancy. The study population comprised pregnant women aged 18–49 years attending ANC services at two healthcare facilities during the study period. Women were eligible to participate if they had a confirmed pregnancy at any trimester, had resided in the facility’s catchment area for at least 3 months, could communicate in isiZulu or English, and provided written informed consent. To avoid duplicate participation, women who had previously completed the survey during the current pregnancy were excluded. Additional exclusion criteria included being younger than 18 years, presenting with a medical emergency or clinical instability requiring immediate care, cognitive impairment or any condition limiting the ability to provide informed consent, postpartum status at the time of recruitment, or any situation where participation could compromise clinical care, as determined by attending healthcare providers.

### 2.4. Sample Size

The sample size was calculated using the single population proportion formula:

n=ε22pZ1−p,

where *n* represents the required sample size, *p* the estimated prevalence, and *ε* the relative precision. Using a 95% confidence level and 5% precision, the minimum required sample size was estimated at 384 participants. After accounting for an anticipated 10% non‐response or incomplete‐data rate, the adjusted sample size was ~428 participants. A final sample size of 420 pregnant women was considered adequate to maintain statistical precision and sufficient power for multivariable analyses while remaining feasible across the two study sites.

### 2.5. Sampling Strategy

A two‐stage sampling approach was used. In the first stage, two CHCs providing ANC services in the eThekwini District were purposively selected: Cato Manor CHC and KwaMashu CHC. We selected these facilities based on three criteria: (i) the facilities had sufficient antenatal patient volume to achieve the required sample size within the available recruitment period, (ii) they served populations representative of the urban and peri‐urban contexts in eThekwini, including communities with substantial social and economic vulnerability, and (iii) their management granted permission to conduct the study following site‐level engagement. Together, the two facilities were considered to provide adequate variation in catchment population, geographic location, and patient demographics within the municipality, while remaining logistically feasible for the planned recruitment period.

In the second stage, a non‐probability consecutive sampling strategy was used in this study. All pregnant women aged 18–49 years attending ANC services at Cato Manor CHC and KwaMashu CHC during the study period were screened for eligibility. Women who met the inclusion criteria and provided written informed consent were recruited consecutively until the target sample size of 420 participants was reached. Consecutive sampling was considered an appropriate approach for a facility‐based study because it enabled systematic enrolment of all eligible women present during the recruitment period. However, as a non‐probability sampling method, it does not eliminate selection bias, and the resulting sample may not be representative of the wider antenatal population; this is acknowledged as one of the study’s limitations.

### 2.6. Data Collection

Data were collected by trained research assistants who approached pregnant women attending routine ANC services and screened them for eligibility for the study using the study’s inclusion and exclusion criteria. Eligible participants were provided with detailed study information in isiZulu or English, and written informed consent was obtained before participation. Data were collected using interviewer‐administered structured questionnaires captured electronically on Android tablets using REDCap software [32]. Research assistants received training on study procedures, electronic data collection, and troubleshooting. The questionnaire collected information on socio‐demographic characteristics, pregnancy‐related factors, oral health status, oral health service utilisation, and food insecurity using the validated eight‐item (FIES‐SM) [33]. As participants were already attending the clinics for routine healthcare services, travel reimbursement was not provided; however, airtime vouchers were offered as a token of appreciation for participants’ time.

### 2.7. Operationalisation of Variables

Household food insecurity was measured using the eight‐item (FIES) Individual Module developed by FAO [9]. Participants were asked whether, over the previous 12 months, they had experienced food‐related constraints due to a lack of money or other resources, such as worrying about food, reducing meal quantity or variety, skipping meals, or going hungry. Affirmative responses were summed to generate a raw score ranging from 0 to 8. Scores were categorised according to FAO‐recommended cut‐offs as food secure (0–1), mild food insecurity (2–3), moderate food insecurity (4–6), and severe food insecurity (7–8). A combined moderate/severe food insecurity variable was additionally created for regression analyses. The eight‐item FIES was developed by the FAO for global application and cross‐country comparability using a Rasch measurement model and has been applied in South African survey research; we used the standard FAO metric without sample‐specific cultural adaptation. In this sample, the eight items showed good internal consistency (standardised Cronbach’s α = 0.82).

Oral health was assessed using items adapted from the WHO Oral Health Questionnaire for Adults (5th edition, 2013). We constructed two oral health outcomes. The first was a 12‐month general oral symptom score (0–4), derived from four binary indicators: tooth or mouth pain, difficulty eating, bleeding gums or loose teeth, and avoidance of smiling, eating, or speaking because of oral problems. Poor oral health was defined as a score≥3. The ≥3 threshold was chosen to identify women with a substantial, multi‐domain symptom burden rather than isolated complaints, prioritising specificity over sensitivity given the self‐reported nature of the measure; because this cut‐off is not externally validated, its robustness was examined across alternative thresholds (≥1, ≥2, ≥3, and ≥4 indicators) in a sensitivity analysis (Table S5). The second outcome was a pregnancy‐onset oral symptom score (0–7) based on symptoms experienced during pregnancy, including bleeding gums, swollen gums, tooth mobility, bad breath, dry mouth, tooth sensitivity, and oral discomfort affecting eating.

Socio‐demographic variables included age, marital status, education level, employment status, household income, household size, and area of residence. Obstetric variables included gestational age, gravidity, parity, and ANC attendance. Oral health behaviours included tooth‐brushing frequency, use of fluoridated toothpaste, and timing of the last dental visit. Dietary and lifestyle variables included meals per day, sugar intake, tobacco and alcohol use, vitamin supplementation, and self‐reported difficulty accessing nutritious foods during pregnancy.

### 2.8. Statistical Analysis

Analyses were conducted in Stata version 19.5 (StataCorp, College Station, TX). Reporting of this study follows the STROBE guideline for cross‐sectional studies. Sample characteristics were summarised using frequencies and percentages. Bivariate associations between candidate predictors and both moderate/severe food insecurity and poor oral health were tested using Pearson’s chi‐square test.

Multivariable associations between food insecurity and poor oral health were modelled using binary logistic regression. The primary model was adjusted for age (<25 vs. ≥25), education (tertiary vs. non‐tertiary), employment status (employed/self‐employed vs. unemployed vs. student), brushing frequency, and last dental visit. We chose reference categories to avoid perfect prediction in sparse cells (no participants aged ≥35 years had poor oral health, and only three women reported less than a high school education). Joint significance of multi‐level variables in the adjusted model was tested using the adjusted Wald test (AWT).

Sensitivity analyses tested four alternative specifications of the food insecurity exposure: (1) categorical FIES (4 levels); (2) continuous FIES score (per one‐point increase); (3) combined moderate/severe binary; and (4) categorical FIES with additional adjustment for difficulty accessing food and meals per day. To address the count nature of the symptom outcomes, negative binomial regression was used to model the 12‐month oral symptom score as a function of FIES and covariates, with results presented as incidence rate ratios.

In an exploratory analysis of whether the association with pregnancy‐onset oral symptoms was attributable solely to pre‐existing general oral health, two negative binomial models were compared. Model A regressed the pregnancy‐onset symptom score on FIES, age, brushing frequency, and trimester. Model B added the 12‐month general oral symptom score (range: 0–4) as an additional covariate. The trimester was further examined using linear trend tests across pregnancy stages and stratified analyses of the FIES–poor oral health association in 1st‐ and 3rd‐trimester subsets. All tests were two‐sided with α = 0.05.

### 2.9. Missing Data

Household income was missing for 33 participants (7.9%); food insecurity and toothbrushing data were each missing for 1 participant. Income was used only in descriptive and bivariate analyses and was not included as a covariate in the adjusted models, which used a complete‐case (listwise) analysis (*n* = 418). To assess whether income missingness was likely to bias the findings, we compared participants with and without income data; those with missing income were more likely to be younger and unemployed but did not differ in food‐insecurity status or the poor oral health outcome. Because income was not a covariate in the adjusted models and its missingness was unrelated to both the exposure and the outcome, complete‐case analysis is unlikely to have biased the reported associations, and a multiple‐imputation robustness check (20 imputations) that imputed income left the primary estimate unchanged.

## 3. Results

### 3.1. Characteristics of the Study Population

Of the 420 pregnant women included, the majority (88.3%) were under 35 years old and 91.4% were single. Most (78.6%) participants had completed high school, and 20.7% had tertiary education. Over half (57.6%) were unemployed, and 44.7% reported a monthly income below R1000 ($60.14). Regarding pregnancy duration, 77.9% were in the second or third trimester, and 37.9% were primigravida. Sample characteristics are detailed in Table 1.

**Table 1:** Sociodemographic, obstetric, and behavioural characteristics of participants (*N* = 420).

Characteristic	*N* (%)
Age	
Mean ± SD range	27.2 ± 6.12
<25	158 (37.6)
25–34	213 (50.7)
>35	49 (11.7)
Area of residence	
Urban	395 (91.0)
Rural	25 (6.0)
Marital status	
Single	384 (91.4)
Married/cohabitating	35 (8.3)
Divorced/separated	1 (0.2)
Education level	
No formal/Primary	3 (0.7)
High school	330 (78.6)
Tertiary	87 (20.7)
Employment status	
Employed/self‐employed	145 (34.5)
Unemployed	242 (57.6)
Student	33 (7.9)
Monthly household income	
<R1000 ($60.14)	173 (44.7)
R1000–R2499 ($60.16–$150.29)	71 (18.4)
R2500–R4999 ($150.35–$300.65)	55 (14.2)
>R5000 (>$300.71)	88 (22.7)
Gestational age	
1st trimester (1–12 weeks)	68 (16.2)
2nd trimester (13–28 weeks)	167 (39.8)
3rd trimester (29–40 weeks)	160 (38.1)
Not sure	25 (6.0)
Gravidity	
1 (primigravida)	159 (37.9)
2	131 (31.2)
3 or more	130 (31.0)
Brushing frequency	
Twice or more daily	365 (87.1)
Once daily	54 (12.9)
Last dental visit	
Within 12 months	34 (8.1)
More than 12 months	154 (36.7)
Never	232 (55.2)

*Note:* Income is missing for 33 participants. Education “no formal education” (*n* = 1) and “Primary” (*n* = 2) collapsed for display. Brushing frequency is missing for 1 participant.

### 3.2. Prevalence of Food Insecurity and Poor Oral Health

Overall, 44.4% (95% CI: 39.7–49.2) of participants reported some level of food insecurity, of whom 20.0% (95% CI: 16.5–24.1) reported moderate or severe food insecurity (Table 2). Poor oral health was reported by 12.6% (95% CI: 9.8–16.2), and 57.0% (95% CI: 52.2–61.7) reported at least one pregnancy‐onset oral symptom, most commonly bleeding gums (35.1%) and dry mouth (34.1%). Prevalence of moderate or severe food insecurity differed significantly across age groups (*p* = 0.005), with the highest proportion, 26.8% (95% CI: 20.5–34.1), among women aged <25 years compared with those aged 35 years or older, 6.1% (95% CI: 2.1–16.5). Food insecurity also differed significantly by monthly household income, with a higher prevalence 27.3% (95% CI: 21.1–34.6) among women earning less than R1000 ($60.14) than among those earning more than R5000 (>$300.71), 11.4% (95% CI: 6.3–19.7). Brushing once daily (*p* = 0.025) and never having visited a dentist (*p* = 0.029) were also significantly associated with food insecurity. No significant associations were observed for residence, marital status, education, employment, trimester, or gravidity (Table 2). Poor oral health differed significantly across age groups (*p* = 0.018), with the highest prevalence (14.6%; 95% CI: 9.9–20.9) among women aged <25 years and no cases among those aged 35 years or older. Brushing once daily was also significantly associated with poor oral health (*p* = 0.007), as was gravidity (*p* = 0.016). No significant associations were observed for residence, marital status, education, employment, income, trimester, or dental visit history (Table 2).

**Table 2:** Prevalence of moderate/severe food insecurity and poor oral health by participant characteristics, with bivariate *p*‐values.

Characteristic	Mod/severe FI % (95% CI)	*p*‐Value	Poor oral health % (95% CI)	*p*‐Value
Overall prevalence	**20.0 (16.5–24.1)**	—	**12.6 (9.8–16.1)**	—
Age group (years)	—	**0.005**	—	**0.018**
<25	26.8 (20.4–34.2)	—	14.6 (9.9–20.9)	—
25–34	18.3 (13.7–24.0)	—	14.1 (10.0–19.4)	—
35+	6.1 (2.1–16.5)	—	0.0 (0.0–7.3)	—
Area of residence	—	0.995	—	0.252
Urban	20.1 (16.4–24.3)	—	12.2 (9.3–15.7)	—
Rural	20.0 (8.9–39.1)	—	20.0 (8.9–39.1)	—
Marital status	—	0.360	—	0.697
Single	20.9 (17.1–25.2)	—	13.0 (10.0–16.8)	—
Married/cohabiting	11.4 (4.5–26.0)	—	8.6 (3.0–22.4)	—
Divorced/separated	0.0 (0.0–79.3)	—	0.0 (0.0–79.3)	—
Education level	—	0.475	—	0.644
No formal/Primary	0.0 (0.0–56.2)	—	0.0 (0.0–56.2)	—
High school	21.5 (17.4–26.3)	—	13.6 (10.3–17.8)	—
Tertiary	15.1 (9.1–24.2)	—	9.2 (4.7–17.1)	—
Employment status	—	0.650	—	0.465
Employed/self‐employed	18.6 (13.1–25.7)	—	13.8 (9.1–20.3)	—
Unemployed	20.7 (16.1–26.3)	—	12.8 (9.2–17.6)	—
Student	21.2 (10.7–37.8)	—	6.1 (1.7–19.6)	—
Monthly household income	—	**0.010**	—	0.195
<R1000 ($60.14)	27.3 (21.2–34.4)	—	12.1 (8.1–17.8)	—
R1000−R2499 ($60.16−$150.29)	14.1 (7.8–24.0)	—	11.3 (5.8–20.7)	—
R2500−R4999 ($150.35−$300.65)	23.6 (14.4–36.3)	—	21.8 (12.9–34.4)	—
>R5000 (>$300.71)	11.4 (6.3–19.7)	—	10.2 (5.5–18.3)	—
Gestational age	—	0.632	—	0.425
1st trimester	23.5 (15.0–34.9)	—	17.6 (10.4–28.4)	—
2nd trimester	18.1 (13.0–24.6)	—	13.8 (9.4–19.8)	—
3rd trimester	19.4 (14.0–26.2)	—	11.3 (7.2–17.1)	—
Gravidity	—	0.364	—	**0.016**
1 (primigravida)	22.8 (16.9–29.9)	—	10.7 (6.8–16.5)	—
2	22.1 (15.9–30.0)	—	19.1 (13.3–26.7)	—
3	12.2 (6.8–21.0)	—	3.7 (1.3–10.2)	—
4 or more	18.8 (10.2–31.9)	—	16.7 (8.7–29.6)	—
Brushing frequency	—	**0.025**	—	**0.007**
Twice or more daily	18.4 (14.8–22.7)	—	11.0 (8.2–14.6)	—
Once daily	31.5 (20.7–44.7)	—	24.1 (14.6–36.9)	—
Last dental visit	—	**0.029**	—	0.237
Within 12 months	11.8 (4.7–26.6)	—	8.8 (3.0–23.0)	—
More than 12 months	14.9 (10.2–21.4)	—	9.7 (6.0–15.4)	—
Never	24.7 (19.6–30.6)	—	15.1 (11.1–20.3)	—

*Note:* Proportions and 95% Wilson confidence intervals are shown. Mod/severe FI = FIES score 4–8 (*n* = 419 with complete FIES data; 1 missing). Poor oral health = three of four 12‐month oral symptom indicators (*n* = 420). *p*‐values from Pearson’s chi‐square test of association between the characteristic and each outcome. Significant *p*‐values (*p*  < 0.05) are shown in bold.

### 3.3. Factors Associated With Poor Oral Health

Table 3 shows risk factors associated with poor oral health. Among the sociodemographic and behavioural characteristics included in the adjusted model (Table 3), food insecurity (*p*  < 0.001) and brushing frequency (*p* = 0.024) were statistically associated with poor oral health. Age (*p* = 0.692), education (*p* = 0.480), employment status (*p* = 0.302), and last dental visit (*p* = 0.944) were not statistically significant. Women with moderate or severe food insecurity were 5.11‐fold (aOR = 5.11; 95% CI: 2.72–9.58; *p*  < 0.001) more likely to have poor oral health compared with food‐secure or mildly food‐insecure women. Each one‐point increase on the continuous FIES score was associated with 44% higher odds of poor oral health (aOR = 1.44; 95% CI: 1.26–1.63; *p*  < 0.001). Women who brushed only once daily were 2.48‐fold more likely to have poor oral health (aOR = 2.48; 95% CI: 1.13–5.43; *p* = 0.024) than those who brushed twice or more daily. Our findings were robust to alternative FIES specifications, adjustment for diet and lifestyle variables, and modelling oral health as a count outcome (Table S1, sensitivity analyses for alternative FIES specifications; Table S2, negative binomial regression of poor oral health on food insecurity). The association was also robust to the definition of poor oral health, with the adjusted odds ratio for moderate/severe food insecurity strengthening monotonically from 2.57 (95% CI: 1.53–4.34) at a threshold of ≥1 symptom to 9.06 (2.82–29.13) at ≥4 symptoms (Table S5).

**Table 3:** Factors associated with poor oral health (*N* = 420).

Variable	Un‐OR (95% CI)	*p*‐Value	aOR (95% CI)	*p*‐Value	AWT *p*‐Value
Food insecurity (FIES)	—	—	—	—	**<0.001**
Food secure	Ref	—	Ref	—	—
Mild	1.76 (0.78–3.97)	0.176	1.72 (0.75–3.95)	0.202	—
Moderate	5.34 (2.52–11.33)	**<0.001**	4.61 (2.12–10.07)	**<0.001**	—
Severe	16.35 (5.52–48.46)	**<0.001**	18.37 (5.78–58.37)	**<0.001**	—
Age group (years)	—	—	—	—	0.692
<25	Ref	—	Ref	—	—
25 or older	0.76 (0.42–1.36)	0.354	0.88 (0.45–1.69)	0.692	—
Education	—	—	—	—	0.480
No tertiary	Ref	—	Ref	—	—
Tertiary	0.65 (0.29–1.43)	0.283	0.72 (0.29–1.78)	0.479	—
Employment status	—	—	—	—	0.302
Employed/self‐employed	Ref	—	Ref	—	—
Unemployed	0.92 (0.50–1.68)	0.782	0.79 (0.40–1.56)	0.494	—
Student	0.40 (0.09–1.82)	0.237	0.28 (0.06–1.45)	0.131	—
Brushing frequency	—	—	—	—	**0.024**
Twice or more daily	Ref	—	Ref	—	—
Once daily	2.58 (1.27–5.21)	**0.009**	2.48 (1.13–5.43)	**0.024**	—
Last dental visit	—	—	—	—	0.944
Within 12 months	Ref	—	Ref	—	—
More than 12 months	1.12 (0.30–4.09)	0.869	1.12 (0.29–4.32)	0.871	—
Never	1.84 (0.53–6.33)	0.336	1.22 (0.33–4.50)	0.768	—

*Note:* The adjusted model includes food insecurity, age, education, employment, brushing, and the last dental visit. McFadden pseudo *R* ^2^ = 0.139; LR *χ* ^2^(10) = 44.08, *p* < 0.001. Significant *p*‐values (*p* < 0.05) are shown in bold.

Abbreviations: aOR, adjusted odds ratio; AWT *p*, adjusted Wald test *p*‐value (joint test of all coefficients for the variable in the adjusted model); Un‐OR, unadjusted odds ratio.

### 3.4. Pregnancy‐Onset Oral Symptoms and the Test for Baseline Confounding

**Figure 2:**
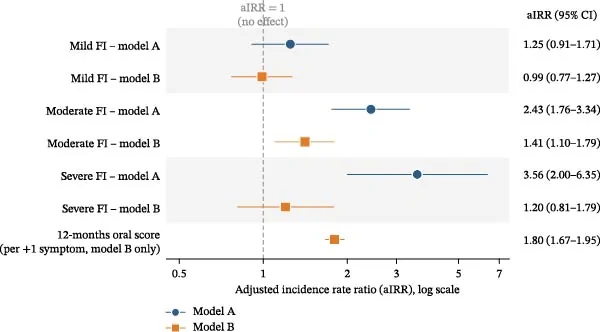
Forest plot of adjusted incidence rate ratios (aIRR) from negative binomial regression of the pregnancy‐onset oral symptom score (range 0–7) on food insecurity (*n* = 393). Model A adjusts for age, brushing frequency, and trimester. Model B additionally adjusts for the 12‐month general oral symptom score (range 0–4). The persistence of the moderate association with food insecurity in Model B (aIRR 1.41, 95% CI: 1.10–1.79, *p* = 0.006) indicates that food insecurity remained associated with pregnancy‐onset oral symptoms after adjustment for women’s baseline oral health. Bold values denote statistical significance (*p*  < 0.05). Full numerical results are provided in Table S4. FI, food insecurity.

To evaluate the effect of food insecurity on oral health during pregnancy, while controlling for baseline differences that women with food insecurity may have at the beginning of pregnancy, we modelled pregnancy‐onset oral symptoms separately (Figure 2; full results in Table S3). In Model A (adjusting for age, brushing, and trimester), moderate food insecurity was associated with 2.4 times as many pregnancy‐onset symptoms as food security (aIRR 2.43, 95% CI: 1.76–3.34). Model B was additionally adjusted for the 12‐month general oral symptom score, which was itself a strong predictor (aIRR 1.80 per one‐symptom increase; 95% CI: 1.67–1.95; *p*  < 0.001). Critically, moderate food insecurity remained independently associated with pregnancy‐onset symptoms after this adjustment (aIRR 1.41, 95% CI: 1.10–1.79, *p* = 0.006), indicating that food insecurity remained associated with pregnancy‐onset symptoms beyond what is explained by women’s baseline oral health status (Figure 2).

Pregnancy‐onset and 12‐month oral symptom scores were both essentially flat across trimesters (linear trend *p* = 0.438 and *p* = 0.495, respectively; Table S4), and the food insecurity–poor oral health association was detectable in both 1st‐ and 3rd‐trimester subsets, supporting both pre‐existing and pregnancy‐period contributions to the overall association.

## 4. Discussion

We aimed to investigate the prevalence and associated risk factors of food insecurity and poor oral health among pregnant women attending public ANC services in eThekwini, KZN.

The prevalence of moderate or severe food insecurity in this cohort was 20.0%, consistent with national South African estimates for 2022 (~16%–19% of households moderately to severely food insecure) [28] and with earlier KZN antenatal cohorts reporting moderate‐to‐severe food insecurity in the mid‐teens [6]. Poor oral health prevalence (12.6%) was lower than 50.2% reporting oral symptoms in a Cameroonian study [34] and 84.9% in a Malaysian cohort [35]. However, differing case definitions limit direct comparison.

The association between food insecurity and poor oral health is consistent with findings among non‐pregnant adults, where food‐insecure adults had higher odds of unmet dental care needs (70.0% vs. 41.0%) [19] and adds to a growing body of evidence across diverse populations [19, 36].

Self‐reported bleeding gums (35.1%), pregnancy‐onset dry mouth (34.1%), and increased tooth sensitivity (19.7%) were common. Although lower than the 60%–75% prevalence of clinically diagnosed gestational gingivitis reported elsewhere, this is consistent with longitudinal evidence that gingival and periodontal changes increase through pregnancy before declining postpartum [37] and with recognised pregnancy‐related changes in salivary composition, fluid balance, and tooth structure [38].

Food insecurity in this population was associated with broader markers of socioeconomic and behavioural disadvantage rather than being a standalone exposure. Never having visited a dentist was more than twice as common among food‐insecure women (24.7% vs. 11.8%), once‐daily brushing was nearly twice as common (31.5% vs. 18.4%), and moderate/severe food insecurity fell from 27.3% among women earning under R1000/month to 11.4% among those earning over R5000/month [16, 39, 40]. These patterns align with South African household data showing food insecurity concentrated in the lowest income quintiles and households without an employed member [28], with international correlates among pregnant women [41] and with a PRAMS‐based analysis in which food‐insecure pregnant women reported worse oral health care experiences, including less preventive care, delayed or foregone dental care, and greater unmet dental needs [20]. Qualitative work has similarly identified economic barriers and low perceived priority of dental care as reasons for non‐attendance [42]. South African national data specifically on brushing frequency remain limited; however, studies from low‐income and rural populations in South Africa and elsewhere in sub‐Saharan Africa have associated lower socioeconomic position and rural residence with less frequent tooth brushing and greater difficulty accessing oral hygiene resources, including affordable fluoride toothpaste [43–45].

After adjustment for the FIES, none of the individual variables associated with food insecurity in univariate analysis (age, education, employment, income, and dental‐visit history) remained independently associated with poor oral health. This suggests food insecurity is a broad indicator of social adversity, more closely related to health‐related behaviours than to socioeconomic status alone, and may largely account for the effects of traditional SES indicators on oral health during pregnancy, consistent with Drumond et al. [46], who found 60% higher odds of dental caries among food‐insecure individuals.

An important and somewhat unexpected finding was the absence of an association between sugar consumption (sugar‐sweetened beverages and sugar‐rich snacks) and poor oral health, in contrast to the strong association with food insecurity and difficulty accessing nutritious food. The single‐item dietary‐frequency measures may be too coarse to capture clinically meaningful variation in a sample where sugar consumption was generally high (39.2% daily sugar‐sweetened beverages and 34.9% daily sugar‐rich snacks), potentially diluting the association. Because dietary intake and biological markers were not directly measured, the pathways linking food insecurity to oral health cannot be adjudicated here. One plausible interpretation, supported by the independent association with difficulty accessing nutritious foods, is that nutritional inadequacy, reduced intake of fruits, vegetables, and protein‐rich foods, leads to deficits in vitamin A, C, D, calcium, and B vitamins required for periodontal tissue integrity, rather than primarily through cariogenic substitution [15]. This is consistent with evidence linking food insecurity to poorer oral health and less access to protective foods [16], including a Ghanaian study in which food‐insecure older adults had higher odds of poor oral health that rose with severity [36]. If so, addressing food insecurity may yield greater oral health benefits through improved access to nutritious foods, integrated into antenatal platforms, than through sugar‐reduction messaging alone.

A persistent question in cross‐sectional pregnancy research is whether poor oral health is a consequence of food insecurity during pregnancy or reflects poorer baseline oral health that predates it, a distinction with different implications for preconception versus antenatal intervention. In our study, food insecurity remained associated with pregnancy‐onset oral symptoms even after adjustment for the 12‐month general oral symptom, consistent with a pregnancy period contribution; however, because this was a cross‐sectional analysis, it cannot establish temporal ordering or exclude residual confounding from women’s underlying baseline oral health. Unlike the earlier PRAMS‐based analysis, which did not distinguish pre‐existing from pregnancy‐emergent conditions [20], the association here was already evident in the first trimester, implying pre‐existing vulnerability, consistent with evidence that oral changes begin early in gestation [38] and that untreated pre‐pregnancy decay predicts offspring caries risk [47]. Together, these findings suggest food insecurity may be implicated in both poorer pre‐pregnancy oral health and additional pregnancy‐onset symptoms, underscoring the value of both preconception and antenatal intervention, ideally initiated in the first trimester.

Clinically, these findings support integrating routine food‐security screening and oral health promotion into ANC: structured oral‐health questions could identify at‐risk women, while pregnancy‐specific education could normalise common symptoms such as dry mouth and sensitivity and offer low‐cost preventive advice. Integrated packages that combine social support, oral health education, and preventive care may particularly benefit food‐insecure pregnant women.

## 5. Strengths and Limitations

Strengths of this study include the use of validated instruments, the Food Insecurity Experience Scale (FIES) and adapted items from the WHO Oral Health Survey, the moderately large sample size, the analysis of both binary and count outcomes with multiple sensitivity analyses for the food insecurity exposure, and an explicit modelling strategy designed to distinguish pregnancy period from baseline confounding contributions.

We recognise several limitations. We did not directly measure pre‐pregnancy oral health, and the pregnancy‐onset symptom analysis provides indirect evidence that the association operates during pregnancy. Still, a longitudinal study would be required for definitive evidence. Participants self‐reported both exposure and outcome, which raises the possibility of common‐method bias. The severe food insecurity group was small (*n* = 17), limiting its precision. The lack of a clinical oral examination limits the ability to differentiate caries, periodontal disease, and other oral pathologies solely from self‐reported symptoms. Because both the exposure and the outcome were self‐reported, non‐differential misclassification is possible. It may have attenuated the observed associations, and self‐reported symptom prevalence is not directly comparable to clinically diagnosed disease. Finally, we restricted the study to women aged 18 years and older, in line with ethical requirements for written informed consent, thereby excluding pregnant adolescents aged 15–17 years. This subgroup may face distinct vulnerabilities, including a higher risk of food insecurity, reduced access to antenatal services, and pregnancy‐onset oral changes that develop against a younger oral health baseline; the findings should not be extrapolated to them without dedicated investigation.

Several further limitations warrant caution in interpretation. Because exposure and outcome were measured simultaneously, the direction of association cannot be established; reverse causation is plausible since poor oral health may impair eating and contribute to household food stress as readily as food insecurity may affect oral health, and residual confounding by unmeasured factors such as underlying poverty and chronic stress cannot be excluded. Participants were recruited from only two purposively selected CHCs using consecutive, non‐probability sampling, which limits external validity and raises the possibility of selection bias, so the estimates should be read as facility‐based rather than population‐representative. Finally, data were collected over a single February–March window; because household food security in South Africa can vary seasonally with agricultural cycles, food prices, and social‐grant timing, the point prevalence reported here may not capture seasonal fluctuation.

### 5.1. Implications for Practice and Policy

Our findings lend support to several practical recommendations. Routine screening for food insecurity should be integrated into ANC, particularly at first booking. Pregnancy‐related oral changes occur early in gestation. The FIES is brief, validated, and easy to administer at routine antenatal visits. Where available, women identified as food insecure should be referred to nutritional support services, including the South African child support grant, any local nutrition voucher, or food parcel programmes and given targeted oral health advice. The strong, independent association between difficulty accessing nutritious foods and oral health (vs. sugar consumption) suggests that interventions that improve access to fruits, vegetables, and protein‐rich foods may yield greater oral health benefits for this population than sugar‐reduction messages alone. Since pregnancy‐onset symptoms are common, basic oral health assessment and education should be part of routine ANC as a low‐cost intervention. Our findings suggest that this gap needs to be addressed as the current South African ANC guidelines do not include routine oral health assessment.

## 6. Conclusion

Household food insecurity was prevalent among pregnant women attending public ANC in eThekwini, KZN, and was significantly and independently associated with poor oral health. This association appeared to be linked more closely to reduced access to nutritious foods than to increased consumption of cariogenic foods; however, the cross‐sectional design does not permit causal or temporal conclusions. These findings support routine food‐insecurity screening during ANC, particularly in the first trimester, to identify women at increased risk of poor oral health who could benefit from integrated nutrition and oral health support. At the national policy level, the findings provide a basis for considering the integration of FIES‐based screening and basic oral health promotion into South Africa’s public ANC system, particularly in high‐burden provinces such as KZN. They also contribute to the global maternal health and food‐security agenda by positioning food insecurity as a cross‐cutting risk marker that may support the integration of nutrition and oral health services within primary healthcare platforms aligned with universal health coverage. Such policies could facilitate earlier identification and support, potentially reducing oral disease during pregnancy and improving maternal nutrition and intergenerational oral health. However, screening policies must be accompanied by adequate resources, staff training, referral systems, food assistance, and accessible dental services to ensure that screening leads to meaningful support rather than simply identifying risk. Longitudinal studies are needed to clarify the direction and mechanisms of the observed association and to evaluate whether integrated interventions improve maternal and child health outcomes.

## Author Contributions

**Sinegugu Shongwe:** conceptualisation, methodology, investigation, formal analysis, data curation, writing – original draft, writing – review and editing, project administration, funding acquisition. **David Seidenfeld:** conceptualisation, methodology, supervision, writing – review and editing. **Themba Ginindza:** conceptualisation, methodology, supervision, writing – review and editing.

## Funding

This research was funded by the American Institutes for Research.

## Disclosure

All authors have read and approved the final version of the manuscript. Sinegugu Shongwe (corresponding author and manuscript guarantor) had full access to all of the data in this study and takes complete responsibility for the integrity of the data and the accuracy of the data analysis. The funder had no role in the study design, data collection, analysis, interpretation, or decision to submit the work for publication.

## Ethics Statement

This study was approved by the University of KwaZulu‐Natal Biomedical Research Ethics Committee (BREC/00009506/2025) and the KwaZulu‐Natal Department of Health Research Ethics Committee (KZ_202511_028). Permission to conduct research at the study sites was obtained from the managers of the eThekwini Health District, Cato Manor Community Health Centre, and KwaMashu Community Health Centre.

## Consent

All participants provided written informed consent. No individual participant data were reported.

## Conflicts of Interest

The authors declare no conflicts of interest.

## Supporting Information

Additional supporting information can be found online in the Supporting Information section.

## Supporting information


**Supporting Information** The following Supporting Information accompany this article: Table S1: Sensitivity analyses for the food insecurity exposure. Table S2: Negative binomial regression of 12‐month oral symptom counts on food insecurity. Table S3: Full numerical results for the pregnancy‐onset oral symptom analysis (Figure 2). Table S4: Linear trend tests and trimester‐stratified analyses of food insecurity and oral health. Table S5: Sensitivity analysis of the food insecurity–poor oral health association across alternative outcome thresholds.

## Data Availability

The data that support the findings of this study are available from the corresponding author upon reasonable request. The data are not publicly available because they contain information that could compromise the privacy of participants who did not consent to public data sharing, in accordance with the ethics approvals governing this study.
