# Supplementary material for: The Double Burden of Food Insecurity and Poor Oral Health Among Pregnant Women in eThekwini, KwaZulu‐Natal
Source: Int J Dent. 2026 Sep 8;2026:9192355. doi: 10.1155/ijod/9192355 (PMC13554725; doi:10.1155/ijod/9192355)
Supplement: Supplementary file 1 [file IJOD-2026-9192355-s001.docx]

**Supplementary Material**

**The double burden of food insecurity and poor oral health among pregnant women in eThekwini, KwaZulu-Natal**

**Supplementary Table S1. Sensitivity analyses for the food insecurity exposure**

| Specification | Adjusted OR (95% CI) | p-value |
| --- | --- | --- |
| **Specification 1: Categorical FIES (4 levels)** |  |  |
| Mild vs food secure | 1.72 (0.75–3.95) | 0.202 |
| Moderate vs food secure | 4.61 (2.12–10.07) | **<0.001** |
| Severe vs food secure | 18.37 (5.78–58.37) | **<0.001** |
| **Specification 2: Continuous FIES** |  |  |
| Per 1-point increase | 1.44 (1.26–1.63) | **<0.001** |
| **Specification 3: Combined moderate or severe** |  |  |
| Moderate or severe vs food secure/mild | 5.11 (2.72–9.58) | **<0.001** |
| **Specification 4: + adjustment for diet single-items** |  |  |
| Mild vs food secure | 1.27 (0.52–3.11) | 0.597 |
| Moderate vs food secure | 2.69 (1.16–6.23) | **0.021** |
| Severe vs food secure | 14.61 (4.04–52.81) | **<0.001** |
| Difficulty accessing food (Yes vs No) | 6.52 (2.79–15.20) | **<0.001** |

*All models adjust for age, education, employment, brushing, and last dental visit (n = 418). Specification 4 additionally adjusts for difficulty accessing food and meals per day (n = 415). Significant p-values (p<0.05) are shown in bold.*

**Supplementary Table S2. Negative binomial regression of 12-month oral symptom counts on food insecurity**

| Predictor | Unadjusted IRR (95% CI) | Adjusted IRR (95% CI) | p-value |
| --- | --- | --- | --- |
| **Food insecurity (ref: Food secure)** |  |  |  |
| Mild | 1.67 (1.23–2.27) | 1.56 (1.15–2.10) | **0.004** |
| Moderate | 2.40 (1.73–3.33) | 2.13 (1.58–2.89) | **<0.001** |
| Severe | 3.38 (2.01–5.67) | 4.21 (2.62–6.78) | **<0.001** |
| Continuous FIES (per point) | — | 1.20 (1.14–1.26) | **<0.001** |
| **Brushing** |  |  |  |
| Once daily (vs ≥2) | — | 1.48 (1.08–2.02) | **0.014** |

*The adjusted model also includes age, education, employment, dental visits, and trimester (n = 393). The negative binomial specification was preferred over Poisson based on overdispersion (LR test of α = 0: p<0.001). Significant p-values (p<0.05) are shown in bold.*

**Supplementary Table S3. Full numerical results for the pregnancy-onset oral symptom analysis (Figure 2)**

| Predictor | Model A: pregnancy-onset symptoms ~ FIES alone, aIRR (95% CI) | Model B: + 12-month oral health score, aIRR (95% CI) | p (Model B) |
| --- | --- | --- | --- |
| **Food insecurity (ref: Food secure)** |  |  |  |
| Mild | 1.25 (0.91–1.71) | 0.99 (0.77–1.27) | 0.948 |
| Moderate | 2.43 (1.76–3.34) * | 1.41 (1.10–1.79) | **0.006** |
| Severe | 3.56 (2.00–6.35) * | 1.20 (0.81–1.79) | 0.366 |
| Continuous FIES (per 1-point ↑) | 1.19 (1.13–1.26) * | — | — |
| **12-month oral symptom score** |  |  |  |
| Per 1-symptom increase (0–4) | — | 1.80 (1.67–1.95) | **<0.001** |

*Negative binomial regression of the pregnancy-onset oral symptom score (range 0–7) on food insecurity (n = 393). Both models adjust for age, brushing frequency, and trimester. Model B additionally adjusts for the 12-month general oral symptom score (range 0–4). The persistence of moderate food insecurity in Model B indicates that food insecurity remains associated with pregnancy-onset symptoms after adjustment for baseline oral health. * p<0.05. Significant p-values shown in bold.*

**Supplementary Table S4. Linear trend tests and trimester-stratified analyses of food insecurity and oral health**

| Outcome by trimester | Mean / % 1st / 2nd / 3rd | Linear trend IRR (95% CI) | p-value |
| --- | --- | --- | --- |
| Pregnancy-onset symptom score | 1.60 / 1.62 / 1.42 | 0.93 (0.77–1.12) | 0.438 |
| 12-month oral symptom score | 0.94 / 0.99 / 0.86 | 0.94 (0.78–1.13) | 0.495 |
| Poor oral health % | 17.6 / 13.8 / 11.3 | — | 0.425 |
| FIES → poor OH (1st trim, n = 68) | Mild OR 8.67* | Severe OR 26.0* | — |
| FIES → poor OH (3rd trim, n = 160) | Mild OR 1.24 | Severe OR 36.5* | — |

*Linear trend tests use trimester as a continuous predictor in negative binomial regression (symptom scores) or Pearson’s chi-square (poor oral health). Trimester-stratified subset analyses are unadjusted logistic regressions of poor oral health on FIES category. * p<0.05.*

**Supplementary Table S5. Sensitivity analysis of the food insecurity–poor oral health association across alternative outcome thresholds**

| Poor oral health threshold | Cases (n) | Adjusted OR, moderate/severe food insecurity (95% CI) | p-value |
| --- | --- | --- | --- |
| ≥1 of 4 symptoms | 194 | 2.57 (1.53–4.34) | **<0.001** |
| ≥2 of 4 symptoms | 115 | 3.52 (2.07–6.00) | **<0.001** |
| ≥3 of 4 symptoms (primary definition) | 53 | 5.11 (2.72–9.58) | **<0.001** |
| ≥4 of 4 symptoms | 16 | 9.06 (2.82–29.13) | **<0.001** |

*Binary logistic regression of poor oral health on moderate/severe food insecurity at each alternative threshold, adjusted for age, education, employment, brushing, and last dental visit, and estimated on the primary analytic sample (n = 418). The ≥3 threshold reproduces the primary estimate reported in the main text. At the ≥4 threshold, no participant in the student employment category reported the outcome, so this category perfectly predicted the absence of the outcome and was omitted, giving n = 386 for that model. The association strengthened monotonically as the definition became more stringent, indicating that the finding is not an artefact of the chosen cut-off. Significant p-values (p<0.05) are shown in bold.*
